# Portable acoustic myography – a realistic noninvasive method for assessment of muscle activity and coordination in human subjects in most home and sports settings

**DOI:** 10.1002/phy2.29

**Published:** 2013-07-10

**Authors:** Adrian P Harrison, Bente Danneskiold-Samsøe, Else M Bartels

**Affiliations:** 1 Department of Veterinary Clinical & Animal Sciences, Faculty of Health & Medical Sciences, Copenhagen UniversityGrønnegårdsvej 7, DK-1870, Frederiksberg C, Denmark; 2 The Parker Institute, Department of Rheumatology, Copenhagen University HospitalBispebjerg and Frederiksberg, DK-2000, Frederiksberg, Denmark

**Keywords:** Acoustic myography, assessment, muscle contraction, muscle function

## Abstract

Muscle sound gives a local picture of muscles involved in a particular movement and is independent of electrical signals between nerve and muscle. Sound recording (acoustic myography) is a well-known noninvasive technique that has suffered from not being easily applicable, as well as not being able to register at sufficient sampling speed. With modern amplifiers and digital sound recording this has changed, and such assessment during movement outside a laboratory setting may be possible. Our aim was to develop a setup for muscle-sound assessment, which could be reliably applied in any local setting. A group of healthy subjects were assessed during standing, stair climbing, walking, and running. Piezoelectric microphones were applied to the skin using contact gel. A digital sound recorder enabled sampling speeds of around 96,000 Hz. Surface electromyography was measured in parallel as a comparison. The recorded signals were assessed and described in terms of signal frequency (Hz) and peak-to-peak amplitude (mV) using Chart software. Bioimpedance of the involved muscles was measured. Sound recording was shown to be an easy noninvasive method for assessment of muscle function during movement with the possibility of being applied in most clinical, sports, and home settings. Muscle sound gives a representation of the work of each muscle group during a complex movement, illustrated here by a step test, which revealed both concentric and eccentric activity. The method in the presented new setup has great potential for assessment of function in patients with musculoskeletal complaints in out-of-clinic settings, as well as in sports.

## Introduction

Sound recording from skeletal muscles, acoustic myography (AMG), has been known as a useful method for assessing muscle force and fatigue for a period of time (Stokes and Blythe 2001), but it is only recently that microphones and contact transducers (piezoelectric devices), as well as recording systems, have become available in a size and of a quality that enables them to be applied to a normal daily setting outside the clinic and the laboratory setting. These new possibilities provide a clinical tool for the assessment of patients with musculoskeletal complaints during daily activities, or assessment of athletes in terms of efficiency in use of muscles. AMG is sometimes confused with the field of mechanomyography (MMG) or accelerometer myography which measure movement vibrations (Hemmerling et al. 2004; Shinohara and Søgaard 2006; Beck et al. 2010; Herda et al. 2010; Tian et al. 2010; Alves and Chau 2011; Qi et al. 2011).With the improved accessibility of piezoelectric crystals, enabling accurate muscle sound recordings transdermally, AMG has had a revival. Many of the earlier AMG studies have been undertaken with a great deal of care (Stokes and Blythe 2001; Bajaj et al. 2002; Hemmerling et al. 2004; Guo et al. 2010), however, a number of limiting factors still remain unresolved, namely the speed of sampling (too low), the size, and weight of the equipment used (too big and heavy – preventing patients to move freely or wander outside of a laboratory environment), noise correction, and finally the development of a better means of sensor/skin contact reducing sensor-air-tissue signal loss.

Recent advances in modern digital sound recorders have enabled sampling speeds at a rate around 96,000 Hz. These new options for improving AMG, in combination with the possibility of having an easily applicable noninvasive assessment method for muscle performance, led us to develop a setup for muscle-sound assessment which can be reliably applied without discomfort for the subject, and not require specialist technical knowledge of the person carrying out the recordings.

With this new AMG technique, this study aimed at evaluating whether the recorded signal could be used to determine coordination, as well as aspects of muscle function, in physically active human subjects with a view to its application in clinical practice.

## Material and Methods

### Subjects

Healthy subjects with no known diseases of the musculoskeletal system were informed of the minimal risks of skin rash (adhesive for surface-electromyography electrodes) in connection with this experimental procedure, and gave their consent. The recorded AMG signals were saved using a code that protected the individual's personal data and identity (e.g., name and date of birth).

Two groups of human subjects were recruited. Group 1 comprised two females who were 24 and 25 years of age, who carried out the following exercises; rise heal-to-toe, walking, stair climbing (step test), and running on the spot at their own chosen speed, all undertaken under clinical laboratory settings. This group was recruited to demonstrate what could be measured in a clearly defined setup, where each movement was controlled. Group 2 was used solely for testing the AMG units during a period of voluntary exercise (running) for a period of 20 min. The individuals in this group were allowed to leave the laboratory setting and run freely on a track around an inner city lake at their own chosen speed. The time between start and finish was recorded, as was the total run distance. This group comprised of four men and six women with ages ranging 33–45 and 23–41 years, respectively. Pulse was measured using a finger sensor (ADInstruments, Chalgrove, Oxfordshire, U.K.), on subjects in group 1 as a way of monitoring heart rate and the effects of physical activity.

### Equipment and measurements

#### Acoustic myography

**Figure 1:**
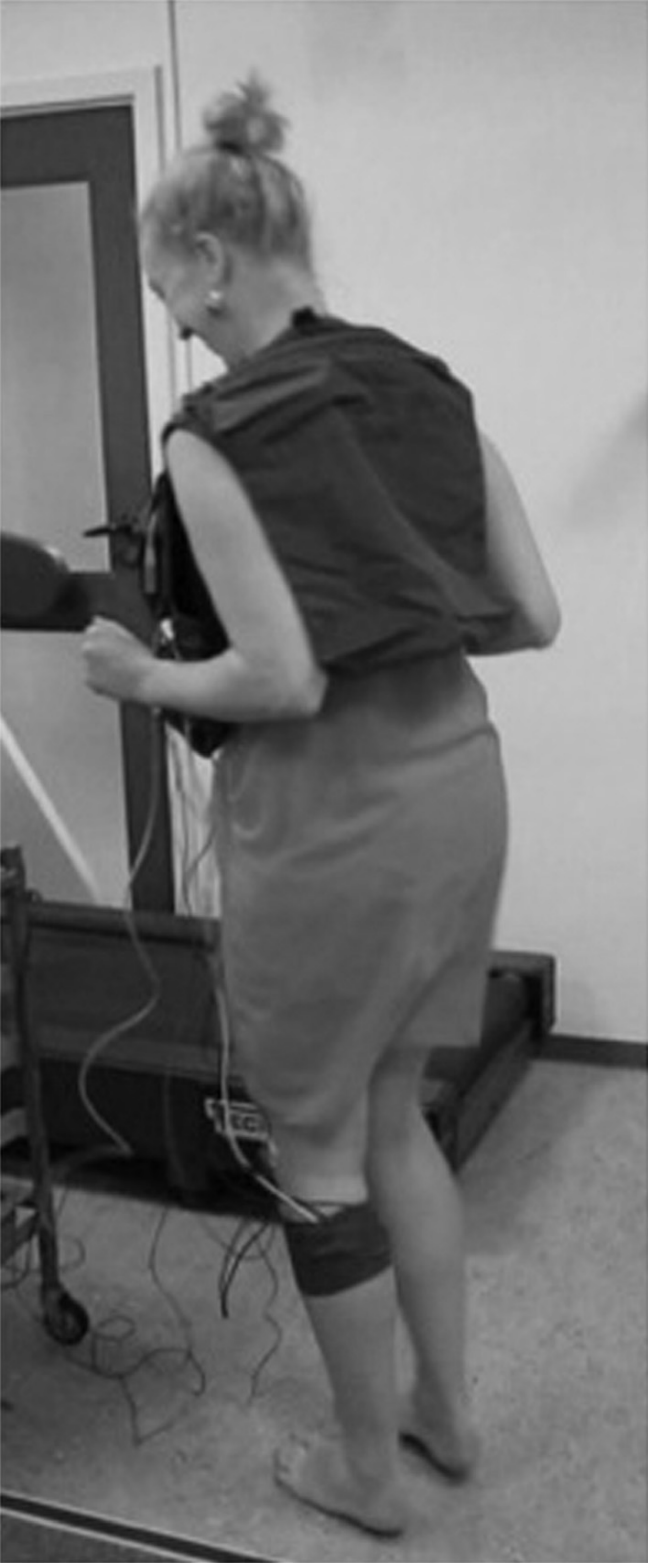
A healthy subject from group 1 running on the spot with both AMG and sEMG electrodes attached to her left m.gastrocnemius. See methods for details.

A purpose-built AMG unit (MyoDynamikApS – http://www.myodynamik.com), capable of recording at a distance of 100 m away from the recording computer by means of a blue-tooth wireless audio transmitter (BTT005; Computer Mester, Herning, Denmark) 44,000 Hz, and with a sampling rate on the recorders of 96,000 Hz, was placed on the lower leg at the point of origin of the lateral m. gastrocnemius of the subjects dominant leg (see Fig. 1). The unit was switched on and recordings of muscle sounds were carried out transdermally.

A commercial contact transducer (piezoelectric) with a resonant frequency range of 0.5–20 ± 0.5 kHz (H10126; BrinckElektronikApS, Copenhagen, Denmark) was covered with Ultra-Sound Gel (BlueScan – Lina Medical ApS, Glostrup, Denmark) to ensure a good connection with the skin above the lateral head of m. gastrocnemius on the leg of the subject and attached with the aid of a self-adhesive bandage (Co-Flex, Andover – Salisbury, MA). The use of the gel further improved the recordings by lowering the lower frequency range to ∼5 Hz.

AMG recordings were taken via a small modified preamplifier (Shure, IL), with sensitivity/filtering capabilities which was placed into a pocket on the subject, and the resulting signal recorded using an acoustic recorder (R-05; Roland, Shizuoka, Japan) at a sampling rate of 96,000 Hz. The recorded signal was sent at 44,000 Hz via a blue-tooth wireless audio transmitter to a receiver connected to a PowerLab/8s A/D converter (ADInstruments, Chalgrove, Oxfordshire, U.K.). The recorded signal was also played back onto a PowerLab/8s A/D converter (ADInstruments, Chalgrove, Oxfordshire, U.K.) at 96,000 Hz and analyzed comparatively with the blue-tooth signal using a Computer with Chart v. 5.5.6/s Software.

The recorded data were analyzed for their frequency and amplitude parameters using Chart software (ADInstruments, Chalgrove, Oxfordshire, U.K.), being sampled as a sound signal. The sound signal derived from the actual contractions of the muscle fibers, which results in mechano or resonance waves transmitted through the tissue to the skin surface (Stokes and Blythe 2001). The central nervous system coordinates muscle function by increasing or decreasing the recruitment and synchronization of motor units (equates to sound signal amplitude), as well by altering the frequency with which active motor units fire (equates to sound signal frequency). Although other factors may influence muscle function, for example, muscle stiffness and hydration (Bartels and Jensen 1982; Hasan and Unsworth 1985). Coordination of the muscle in terms of physical activity could be assessed by periods of active/inactive function, and the relative duration of each period (Harrison et al. 2006).

There was no health issues associated with this technique, recording via a microphone.

#### Postprotocol measurements of the AMG signal

Following the per-protocol measurements the question as to whether the recorded AMG signal represented wholly or partially noise not derived from the active skeletal muscles was assessed. To answer this methodological question, AMG signals were obtained from the m. biceps femoris during an arm lift without a weight, an arm lift including a weight, and the signal obtained when the arm was passively lifted by another individual.

#### Surface electromyography

The surface electromyography (sEMG) recordings were obtained as a validation of, and in parallel with the AMG in group 1, following the guidelines laid out in the *European Recommendations for Surface ElectroMyoGraphy* (Hermens et al. 1999). The recorded surface EMG signal was assessed as described previously in terms of signal frequency (Hz) and peak-to-peak amplitude (mV), using Chart analysis software (ADInstruments, Chalgrove, Oxfordshire, U.K.) (Harrison et al. 2006).

A double differential electrode configuration, with electrodes (N-00-S & R-00-S; Blue Sensor, Medicotest A/S, Ølstykke, Denmark) was adopted (Harrison et al. 2006). sEMG recordings were taken via an ML 132 amplifier connected to a ML780 PowerLab/8s A/D converter (ADInstruments, Chalgrove, Oxfordshire, U.K.) with a further connection to a MacBook Air with Chart v. 5.5.6/s Software, Peak Parameters and Spike Histogram extensions. Input impedance was 200 MΩ differential, and a high- and a low-pass filter of 3 Hz and 500 Hz, respectively, were used. Sampling speed was set to 100,000 per second, which is way above what is needed in terms of the Nyquist frequency (Diniz et al. 2002).

Measurements, taken in parallel to the AMG recordings, were via two electrodes placed on the medial head of m. gastrocnemius of the nondominant leg of the subjects.

#### Bioimpedance analysis

In group 1, the health of the involved muscles was tested by recording bioimpedance (BIA) in the standing position, and kept free of all metal surfaces (Cai et al. 2000; Salinari et al. 2002). A precisely determined anatomical area of the subject was localized, spanning the Achilles tendon/m. gastrocnemius junction to the point of origin of the medial and lateral heads of m. gastrocnemius. Four Blue Sensor (MedicotestAS, Ølstykke, Denmark) self-adhesive electrodes (1 × 3 cm) were placed onto the sites.

A BIA unit (C-guard, Danmeter, Odense, Denmark; 200 μA current, 1.5 and 100 kHz) was subsequently attached to the electrodes and recordings of the resistance (R) were made. For all electrode placements, the outer two electrodes provided the electrical field, and the inner pair was sensing. Measurements were derived for Impedance at 1.5 kHz (low) and 100 kHz (high). For further details regarding the BIA technique see Ivorra (2003).

### Statistical analysis

Data are presented as mean ± SD. Differences between means were tested for statistical significance with the use of GraphPadInstat 3 for Mac (Version 3.0b, 2003; GraphPad Inc., La Jolla, CA), with an additional test for Gaussian normal distribution, to justify the use of the mean. Differences between means showing a *P* value >0.05 were considered nonsignificant.

## Results

### Group 1 – Validation of method

The BIA resistance for m. gastrocnemius of subjects in group 1 (five repeat measurements per subject and per frequency) yielded values of between 69.8 and 70.1 Ω at low frequency (current passing primarily via the extracellular fluid), and 52.5–56.9 Ω at high frequency (current is primarily forced to pass through the cells) – for further details see Ivorra (2003). These values are typical for healthy individuals with no muscle damage (Nescolarde et al. 2013).

**Figure 2:**
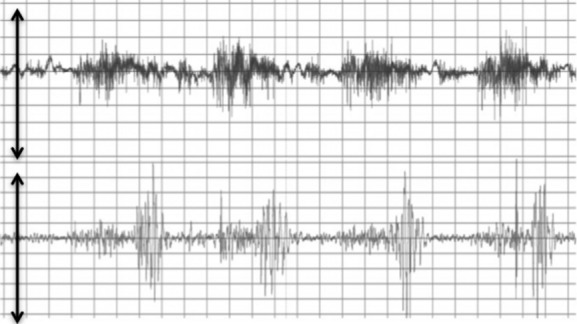
A typical AMG and sEMG trace obtained from one of the healthy subjects in group 1. Upper trace = sEMG and Lower trace = AMG recordings from m. gastrocnemius during a heal-to-toe rise and fall form of physical activity. Note that the sEMG signal starts a little prior to the AMG signal, commencing with the neuromuscular junction contribution to the sEMG signal, followed by the compound muscle action potential (CMAP) contribution as the muscle fibers subsequently depolarize and contract. Likewise, of interest, is the tendency in this trace for the concentric part of the contraction to result in a weak AMG signal, while the eccentric phase of the contraction gives a much stronger AMG signal. Scale – upper panel = ∼8 μV and lower panel = ∼0·16 mV. *x*-axis – each square = 1 sec.

**Figure 3:**
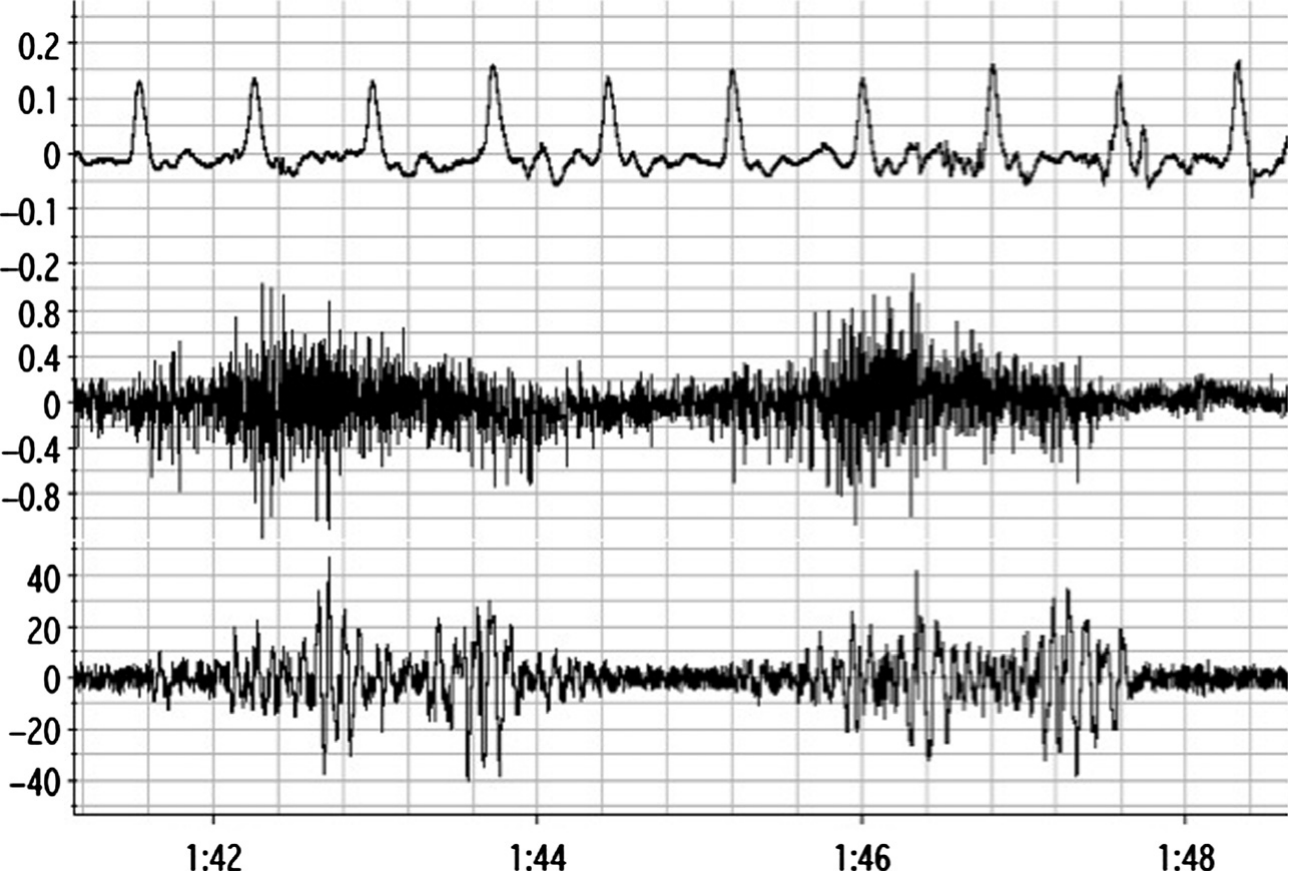
A typical AMG and sEMG trace obtained from one of the healthy subjects in group 1. Upper trace = pulse, middle trace = sEMG, and lower trace = AMG recordings from m. gastrocnemius during a period of stair climbing as a form of physical activity. Note that the sEMG signal starts a little prior to the AMG signal, commencing with the neuromuscular junction contribution to the sEMG signal, followed by the compound muscle action potential (CMAP) contribution as the muscle fibers subsequently depolarize and contract. The AMG signal shows the more complex type of contraction involved in this form of activity. It reveals the coordination that is required with an initial concentric contraction (leg lift), followed then by an eccentric contraction (foot touching the stairs), and a repeating sequence of concentric and eccentric contraction associated with balance as the body's weight is shifted onto this leg enabling the free leg to be raised to the level of the next step. Scale *y*-axis = μV, *x*-axis = minutes:seconds, for example, 1 min 42 sec = 1:42.

The subjects in group 1 were recorded simultaneously for their sEMG signal (medial m. gastrocnemius) and their AMG signal (lateral m. gastrocnemius), while also monitoring pulse. A typical trace for these recordings can be seen in Figure 2, where the subjects were performing heal-to-toe rise physical activity, which recruits the m. gastrocnemius both in terms of concentric (rise) and eccentric (fall) contractions. Figure 3 shows a recording from stair climbing, which is representative of the six repeat measurements taken per subject, and also very similar between subjects.

The subjects in group 1 were also asked to run on the spot for ∼2 min, and during such physical activity the AMG signal was found to have a mean frequency of ∼60 Hz, values that were very similar to those obtained for the runners in group 2 who were deemed to be relatively inefficient.

### Group 2 – AMG during movement in a wireless setting

The runners in group 2 were calculated as having an average run speed, based on a 20 min run over a known distance giving a speed of 11.0–16.5 km per hour for the men and 6.9–13.5 for the women. The AMG average frequency varied greatly between these individuals, but the AMG signal average amplitude, indicative of muscle fiber recruitment, was found to be very similar both at the start and finish of the 20 min run; 0.17 ± 0.01 and 0.17 ± 0.04 mA (mean ± SD), respectively (*P* > 0.05).

**Figure 4:**
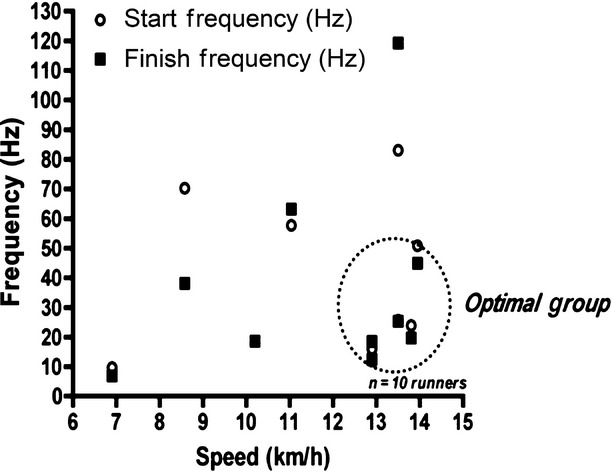
A graph of AMG signal frequency (Hz) against average running speed (km/h) for the 10 healthy subjects in group 2. Each runner is characterized by their running speed. An open and closed symbol aligned vertically indicates the start and finish frequencies for an individual runner. Those runners with an identical start and finish frequency will appear to have overlapping symbols. The AMG recordings obtained from the runners in group 2 (m. gastrocnemius) reveal a wide spread in terms of AMG signal frequency (Hz), with the optimal runner group using a very low AMG frequency (fewer muscle fibers recruited, that is, better energy conservation: circled symbols) to obtain a fast speed. This figure also highlights individual runners that have a good speed, but have a high AMG frequency (inefficient use of energy by recruitment of too many muscle fibers) and a few runners that have almost walked round the course using very few fibers and attaining a very low average speed.

AMG signal frequency (Hz) is seen against average running speed (km/h) for the subjects in group 2, Figure 4. The data reveals a wide spread in terms of AMG signal frequency (Hz), with the optimal runner group using a very low AMG frequency (fewer muscle fibers recruited, i.e., better energy conservation) to obtain a fast speed. Recalling that the runners had a very similar AMG amplitude (fiber recruitment), some of the runners showed a good speed, but had a high AMG frequency, that is, inefficient use of energy resulting from a more frequent activation of the recruited muscle fibers. In contrast, a few runners almost walked round the course with a very low average speed and were therefore activating very few fibers at any point in time (Fig. 4).

### Methodological validation

**Figure 5:**
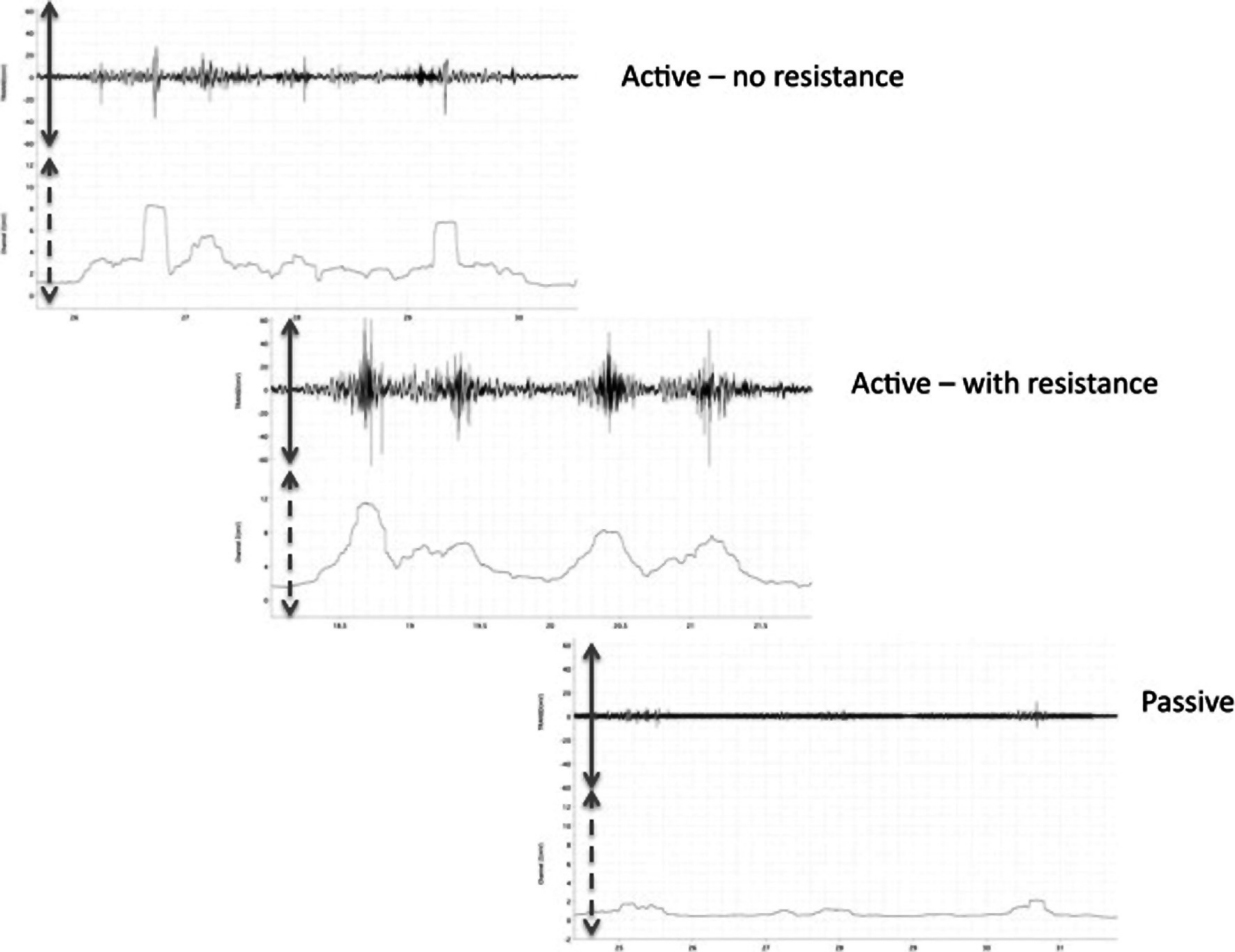
A graph of the AMG signal from m. biceps femoris showing the response to an arm lift without a weight (upper panel), an arm lift with a weight (middle panel), and the signal obtained when the arm is passively lifted by another individual (lower panel) – note the increase in AMG signal with a weight versus no weight, and the lack of AMG signal when no fibers are recruited but the arm is passively lifted.

Figure 5 demonstrates the AMG signal difference between the use of an active muscle, a weight bearing active muscle, and a muscle that was passively lifted by another individual. It is clear from the recordings that the passive lift does not create a signal, and with an increase in weight bearing there is an incremental increase in AMG signal. We can therefore discard the query that the AMG signal is wholly or partially indicative of background noise.

No difference between the blue-tooth wireless audio AMG signal and the locally recorded signal obtained from the acoustic recorder were observed.

## Discussion

Our findings show that with recent technological developments in both piezoelectric microphones and digital sound recorders, AMG is not only an accurate method to assess muscle function and coordination, it can also become wireless.

Our recordings add to the knowledge obtained from previous attempts to record muscle sounds during contractions in human subjects (Stokes and Blythe 2001; Bajaj et al. 2002; Guo et al. 2010). However, earlier studies were neither able to record from freely moving subjects nor was their sampling rate always sufficient enough to accurately monitor high-frequency muscle sounds which originate from muscle contractions. Such muscle sounds are inherently of very low amplitude, and at low sampling rate they become undetectable from background noise. The earlier piezoelectric microphones were also very fragile, which made it difficult to apply them during muscle work.

In the earlier studies it was possible to measure amplitude (e.g., fiber recruitment = strength of contraction) using AMG (Stokes and Blythe 2001; Bajaj et al. 2002; Guo et al. 2010). The recent advances in measuring equipment demonstrated here enables us to record at a frequency which assures accurate assessment of low- and high-frequency sound parameters. This contrasts with previous studies, where the loss of high-frequency/low amplitude sound signals resulted in a nonrepresentative recording with part of the signal, due to recording at a set frequency bandwidth, being filtered out.

With our setup we wished to use the recent technological advances to demonstrate the strength and potential of the AMG technique, and to validate the recorded sound signal against the more established sEMG method which detects electrical depolarisations of both the neuromuscular junction and individual muscle fibers. We also assessed subjects in terms of BIA in order to be certain that we were recording from not only healthy individuals, but also a healthy muscle with no hidden defects.

When measured together with sEMG (Fig. 2), the AMG clearly follows the sEMG pattern. It is also clear that high-frequency muscle sounds can be recorded using AMG (they just have small amplitudes, making a high recording speed essential for accuracy). Earlier studies would sadly not have been able to see these AMG signals (Stokes and Blythe 2001; Guo et al. 2010), as the recording systems used were not sensitive enough in terms of signal frequency. While EMG and MMG have been simultaneously recorded and compared, that is, Orizio et al. (2003), we are not aware of AMG studies with simultaneous recordings of sEMG.

Our AMG recordings (see Figs. 2 and 3) clearly reveal both the concentric and eccentric phase of contraction (demonstrated here by standing on toes and the more complex movement of climbing stairs), and provide an insight into coordination. This highlights the potential of this technique as a means of separating different muscle groups during a complex movement. During the concentric phase of muscle work, the frequency of the sEMG signal is quite high (93 Hz) and the amplitude quite large (1.25 mV). This corresponds to a high-frequency (30 Hz) AMG muscle sound signal with a small amplitude (17 mV), indicating very fast but small physical movement of the active muscle fibers. Then, during the eccentric phase, the sEMG signal reduces amplitude (0.79 mV) and frequency (50 Hz), enabling the previously contracted muscle to relax and lengthen. The AMG muscle sound signal at this stage has a relatively large amplitude (28 mV) and lower frequency (20 Hz), which is representative of a change toward activation of slow-twitch and intermediary fibers, something that is known to occur with a transition from concentric to eccentric contraction (Nogueira et al. 2013). It is known from physics that a low-frequency oscillation of a fiber results in a relatively large amplitude signal (dB magnitude) compared with a high-frequency oscillation where a much smaller amplitude (dB magnitude) arises (Holm and Toiviainen 2004).

Concentric muscle work requires increasing recruitment of muscle fibers and their stimulating frequency to produce the force needed, while during eccentric contraction the muscle is initially exposed to a reduction in stimulating frequency, allowing the muscle to lengthen once again. It is at the point of transition from concentric to eccentric that the muscle should ideally gradually and slowly reduce force, thereby preventing force-related damage to both muscle fibers and connective tissue alike. This movement also needs to be coordinated to avoid high velocity movement during eccentric muscle work, something that is known to induce muscle damage (Nogueira et al. 2013).

Our AMG signal shows an increase in amplitude during the eccentric cf. concentric phase, and this would indicate an increase in fiber recruitment – probably through activation of the muscle spindle receptors (extrafusal fibers) (De Luca and Kline 2012). This is a response to the increase in muscle length and, apart from inducing contraction of the muscle spindles, it must also induce recruitment of additional muscle fibers to take up the strain of the eccentric muscle work.

When looking at Figure 3, it is worth noticing that walking up and down a set of stairs is a compound movement consisting of an extra concentric – eccentric movement necessary in order to maintain balance. This may be useful in the assessment of subjects with balance and stair-climbing problems, where each part of the movement can be analyzed, and a decision whether to act to correct a movement pattern subsequently made.

When looking at the runners in group 2, it is demonstrated that AMG can be measured during high physical activity. Furthermore, it is possible to consider how efficient each subject is able to use their muscles. This may be applicable in an assessment of athletes and their limits of achievements. What is remarkable is that some of the runners were as inefficient as the test subjects in group 1, who were wired up with EMG-electrodes and had a more limited range of movement.

A physiological issue with sEMG recordings is that the signal is a compound of both the neuromuscular junction as well as of active muscle fibers, making it difficult to assess whether a high frequency or amplitude is mostly comprised of neural signals or actual muscle fiber depolarizations. The AMG signal, on the other hand, is comprised of the mechano-sound waves arising from muscle fiber contractions. As a consequence, it does not represent or include neuromuscular junction signals. It is in this way a clearer muscle activity signal and can thus be used to assess when a muscle is active or inactive. AMG can therefore determine when a muscle or muscle group is being misused, leading to pain and injury.

While far above the requirements for the Nyquist frequency, our very fast sampling speed enables the possibility to analyze recorded data in a much more precise fashion. With for example, 96,000 samples a second, even very high-frequency AMG signals can be analyzed with a great degree of accuracy allowing us to confidently identify such small amplitude sound waveforms from background noise. The validation of this apparatus (Fig. 5), showing minimal extraneous noise interference, makes our update of an old method a promising assessment tool for muscle function.

A major technical advantage of the AMG technique compared to sEMG is that sweat, produced by subjects during periods of exercise, does not interfere with the recorded signal, but in fact serves to improve it – less tissue to recorder signal loss. Often sEMG electrodes become unstable and begin to move once a subject starts to sweat, and this electrode-skin contact loss leads inevitably to recorded artifacts and analysis errors.

Concerning interfering noise components, AMG recordings would be sensitive to such environmental noise as clothing contact – which can easily be avoided, very loud sounds (e.g., shouting, door slamming) but our runners had no such issues in the town environment, and the subjects recorded in a clinical setting were not subject to such extraneous environmental noise – in most settings this will not therefore be a problem. Furthermore, the use of Co-Flex also serves as a noise filter and the gel preferentially enhances the sound transfer in a unidirectional manner from the muscle to the microphone. Additionally, these microphones are in fact built as primarily unidirectional recorders with a sounding board on one side and piezoelectric ceramic crystals on the other. The AMG technique can, however, be affected by electromagnetic interference from, for example, old-unshielded electric treadmills, which almost totally disrupt the signal to the point of over-loading the recording units. This type of interference is so apparent that it can easily be avoided.

A substantial advantage is also that AMG now can be measured in a wireless system, making it possible to carry the small measuring devices around when carrying out activities of daily living in ones own home or when participating in sports. It may in this connection be possible to measure muscle function with AMG during an Assessment of Motor and Process Skills (AMPS) test (Fisher and Jones 2010) in function laboratories to supplement the information on physical ability with direct muscle function.

## Conclusion

Overall, the new portable AMG technique is a promising noninvasive method to assess movement in most settings.
